# Compliance With NICE Guidance for Cervical Spine Trauma Imaging: A Single Centre Retrospective Evaluation

**DOI:** 10.1002/jmrs.70105

**Published:** 2026-07-02

**Authors:** Nikoll Walker, Jas Sawhney, Beverly Snaith

**Affiliations:** ^1^ Radiology Doncaster and Bassetlaw Teaching Hospitals NHS Foundation Trust, Doncaster Royal Infirmary Doncaster UK; ^2^ Faculty of Health & Social Care University of Bradford Bradford UK; ^3^ Radiology Mid Yorkshire Teaching NHS Trust Wakefield UK

## Abstract

**Introduction:**

In 2016 the National Institute for Health and Care Excellence (NICE) published guidance in England recommending computed tomography (CT) as the first line imaging for cervical spine (C‐spine) injury in adults. However anecdotal evidence suggests that X‐rays are still being performed. The aim of this evaluation was to determine the imaging referral patterns following C‐spine trauma and compare with national guidance.

**Methods:**

Within a single English organisation emergency department patient attendance data, X‐ray and CT referral criteria and acquisition information for patients ≥ 16 years undergoing imaging of the C‐spine was collated from a 12‐month period (01.01.23–31.12.23).

**Results:**

The cohort contained 302 females and 301 males, with a median age of 55 years. According to the clinical information 12.1% (*n* = 73/603) of the patients had no risk of C‐spine injury and therefore should not have required diagnostic imaging. Almost two thirds of patients (*n* = 395/603; 65.5%) had first line CT imaging consistent with national guidance. Of those where imaging was justified, 135 patients had the incorrect initial diagnostic test (X‐ray as opposed to CT) with 26 also having a subsequent CT. The quality of all of the X‐rays were reviewed and only 39.5% (*n* = 73/185) of cases were diagnostic.

**Conclusions:**

In 2023, the organisation was 65.5% compliant with the guidance, with a number of patients referred for unjustified imaging examinations and/or the wrong initial imaging modality. The consequences of this included treatment delays, prolonged immobilisation, over‐irradiation and impact upon resources, along with potentially missed pathology due to inconclusive X‐rays.

## Graphical abstract

This study found that only two‐thirds of patients were managed in accordance with national guidance for C‐spine trauma, with younger patients more likely to have X‐rays rather than CT. Inappropriate imaging carries radiation dose implications and delays in care with subsequent risks, particularly where immobilisation is utilised.

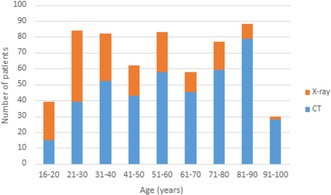

## 1 Introduction

Cervical spine (C‐spine) injuries are uncommon, but where the spinal cord is involved they can be devastating, resulting in serious neurological damage, or even death [1]. Exaggerated flexion, extension and rotation along with abnormal compression can together, or individually, result in significant injury due to the complex anatomy. Every year in the United Kingdom (UK) up to 800 people will be diagnosed with a traumatic spinal cord injury (SCI) [2], with thousands more sustaining an isolated fracture or dislocation without concomitant SCI [3, 4]. The World Health Organization (WHO) [5] state that most SCIs are preventable as they are attributable to falls, road traffic injuries or violence. Globally, there is a male predominance [5] and a bimodal distribution, with higher incidence seen in young adults and the elderly [6]. If there is any suspicion of C‐spine injury then immediate full in‐line immobilisation is essential to protect the spine [7, 8, 9].

It is recognised that diagnosis of a C‐spine injury is reliant on imaging as a supplement to clinical examination [10], and cross‐sectional imaging is increasingly used as an adjunct or replacement for X‐rays [11]. National guidance on the management of patients with a C‐spine injury exists in a number of countries [12], with the body responsible for clinical effectiveness and guidelines in England, the National Institute for Health and Care Excellence (NICE), publishing their NG41 guidance *Spinal injury: assessment and initial management* [13] in 2016. Its objective was to decrease morbidity and mortality through improvements in urgent and emergency care of such injuries.

To increase the appropriate use of imaging, NICE recommended a robust clinical assessment utilising the Canadian C‐Spine Rule (CCR) [14] to identify those at risk of injury. The CCR is an incremental algorithm where the initial assessment of age, mechanism of injury, and paraesthesia can independently justify referral for imaging [12]. Only if none of these factors are present is range of movement considered, and in the absence of risk factors, imaging is not advised [10, 13]. NICE also went on to advocate for computed tomography (CT) as the first‐line imaging investigation in adults aged 16 based on the poor sensitivity and specificity of X‐rays. Different guidelines exist and in their review of three key publications [13, 15, 16], Gesu et al. acknowledged their inconsistency on a number of factors but confirmed they all agree on CT as a primary imaging tool [12].

Despite the publication of NG41 [13] it is acknowledged that X‐rays are still being performed within the UK [11, 17], with an acknowledgement that patients may undergo a subsequent CT scan as a result of poor quality or inconclusive X‐rays or ongoing clinical suspicion. Importantly, X‐rays are still advocated in contemporary articles describing the early management of injuries based on their low cost, speed [11] and ready availability, despite recognised challenges in image interpretation, particularly in the elderly [18]. This leads to questions about the validity of the initial imaging and concerns regarding radiation dose, both factors relevant to radiographers. The aim of this single centre evaluation was to examine referral patterns for imaging of patients following C‐spine trauma thereby stimulating wider debate and review of current practice within emergency care services.

## 2 Method

This retrospective evaluation took place at a multi‐site NHS Trust in the North of England, comprising two emergency departments (EDs) and a minor injury unit. Institutional ethical approval was obtained prior to data collection as the project was undertaken as part of a postgraduate academic award. Ethical approval was given by University of Bradford (ref 23039456) and the NHS Trust (ref 0016/2024/RAD/MI/CB).

### 2.1 Data Collection

The inclusion criteria comprised adults (aged 16 years and over) referred by the ED for primary C‐spine imaging (X‐ray or CT) following isolated trauma to the head and/or neck. Those without a history of trauma, known to have a spinal fracture or polytrauma were excluded. Anonymised data regarding referrals for C‐spine were extracted over a 12‐month period (01.01.23–31.12.23) from the radiology information system (RIS) (Zillion; Rogan‐Delft; The Netherlands), including the date and time of attendance, age and gender. Data on mechanism of injury (MOI) and symptoms were collated from the ED notes. The referrer role, patient transportation method, date, time and content of the imaging report, radiation dose (dose area product (DAP) for X‐rays and dose length product (DLP) for CT) were gathered from the RIS. Information regarding the number and type of X‐ray projections undertaken was extracted from the Picture Archiving and Communication System (PACS) (Fujifilm Synapse; Japan). In addition, any comments documented by ED staff or radiographers related to the referral, justification or conduct of imaging were transcribed verbatim.

Review of the referral against NICE guidance [13] and the adequacy of X‐rays was performed on a diagnostic PACS workstation by the first author, a radiographer with 20 years of independent image interpretation experience. At the study site a complete trauma X‐ray examination of the C‐spine includes a minimum of three projections: the anteroposterior (AP) which demonstrates C3–C7, the peg, which displays C1 and C2 through the patient's open mouth and the lateral that shows the entire C‐spine. A binary decision was made regarding image quality, with examinations being categorised as *adequate* or *inadequate* based on visualisation of all relevant anatomy, both anatomically and due to the exposure, and an absence of rotation. For the study, a pragmatic decision was made that the cervico–thoracic junction had been visualised on the lateral projection when the entire C7 vertebral body was demonstrated.

### 2.2 Data Analysis

Comparison of demographic, mechanism and symptom data to the CCR [14] enabled risk stratification of injuries. Descriptive analysis of patient data was undertaken in Microsoft Excel with subsequent statistical analysis undertaken using an online statistics calculator (www.socscistatistics.com) with a significance level of 0.05. Mann–Whitney *U* and Chi squared tests enabled comparison of groups in relation to age, gender, initial imaging modality, immobilisation, cervico–thoracic junction visualisation and the trauma‐related findings. Referrer profession and grade were analysed to determine any pattern of modality utilisation amongst the different groups.

## 3 Results

**FIGURE 1:**
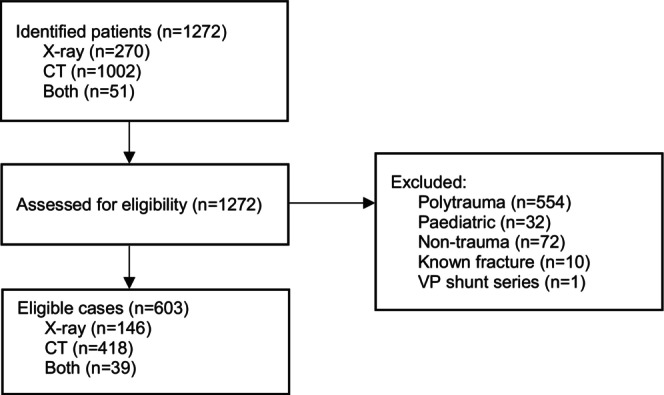
Review process against the inclusion and exclusion criteria.

Within the study period there were 1272 patients referred by the ED for 1323 C‐spine imaging examinations, with 603 meeting the inclusion criteria, the remainder excluded predominantly as a result of polytrauma (Figure 1). The majority had a CT scan (*n* = 457/603; 75.8%), with 39 of these having X‐rays first.

**FIGURE 2:**
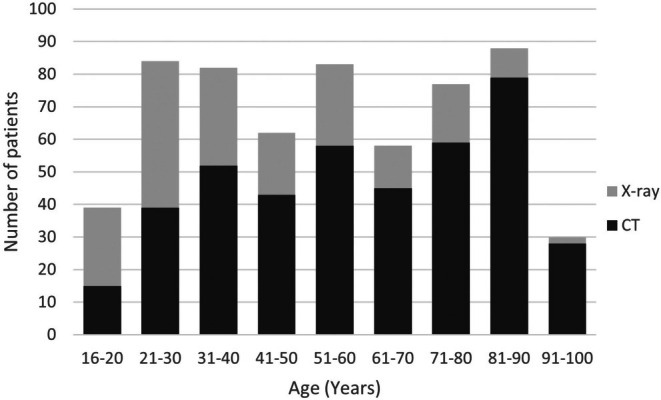
Initial imaging modality compared to the age distribution of patients.

Of the 603 eligible cases there were a similar number of females (*n* = 302) and males (*n* = 301), with a median age of 55 years (range 16 to 96 years) and 42.0% were over the age of 60 (*n* = 253/603). The older patients were more commonly female (female median = 59 years vs. male median = 51 years; *z* = 3.245; *p* < 0.05). Figure 2 demonstrates that the older patients were more likely to be referred for a CT scan as the initial imaging examination (CT average 60 years vs. X‐ray average 43 years; *z* = 3.245; *p* < 0.05).

**FIGURE 3:**
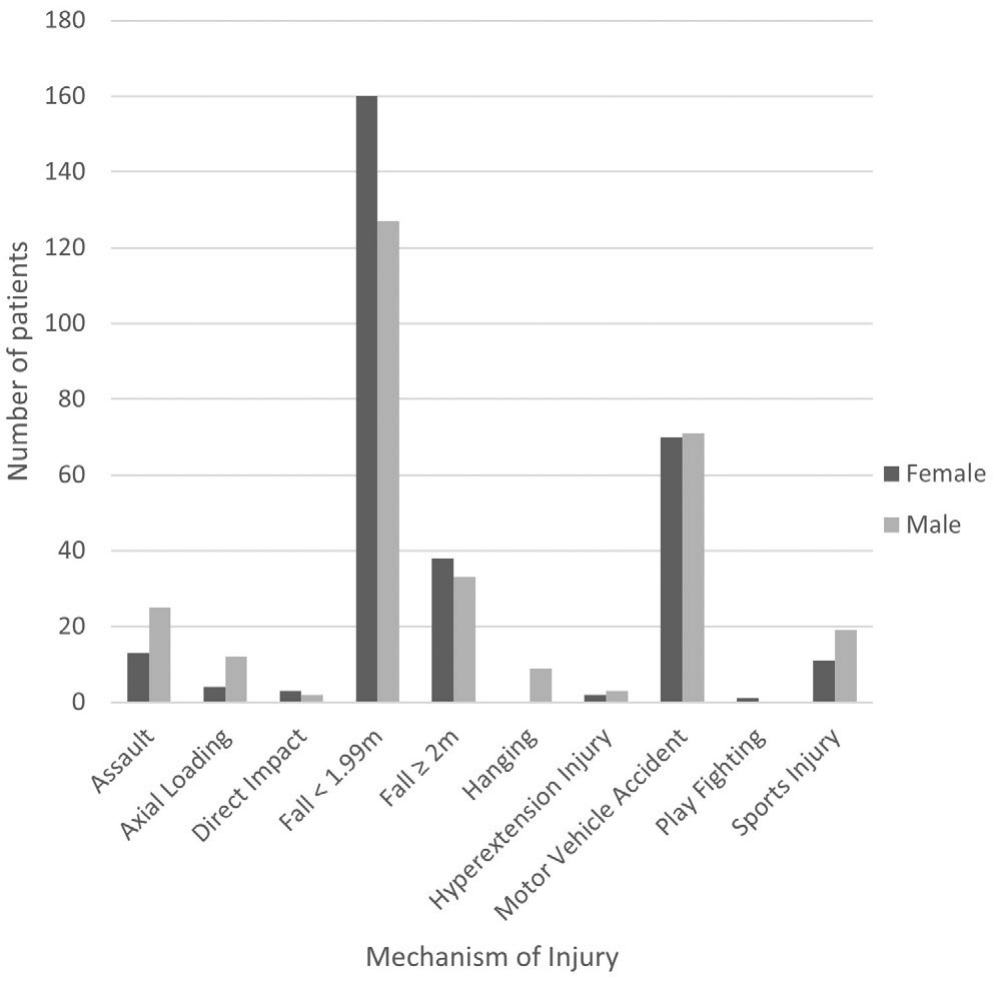
Gender specific mechanism of injury data.

The most common MOI was a fall, including 95.7% (*n* = 222/232) of patients aged > 65 years, almost all being under 2 m (*n* = 202/222; 91.0%). In patients aged ≤ 65 years the MOI included motor vehicle accident (MVA) (*n* = 126/350; 36.0%), falls (*n* = 122/350; 34.9%) with over half (*n* = 73/122; 59.8%) being less than 2 m, assaults (*n* = 37/350; 10.6%) and sports injuries (*n* = 30/350; 8.6%). Comparison of MOI to gender is shown in Figure 3.

Of the cohort 69.3% (*n* = 418/603) had CT as the initial imaging, with no specific pattern amongst medical staff, regardless of grade, and nurse practitioner referrers. There was a significant association between spinal immobilisation and the modality (*χ* ^2^ = 123.694; *p* < 0.05), including 94.3% (*n* = 394/418) of CT scans and only 57.3% (*n* = 106/185) of X‐rays. Of the 79 patients who presented for X‐ray without immobilisation, 10 (12.7%) were on a trolley, with the remainder in a wheelchair (*n* = 18; 22.8%) or walking (*n* = 51; 64.6%). A number of those without immobilisation were considered, on the basis of the demographics and/or history, to be at high risk of C‐spine injury (*n* = 35/79; 44.3%).

**FIGURE 4:**
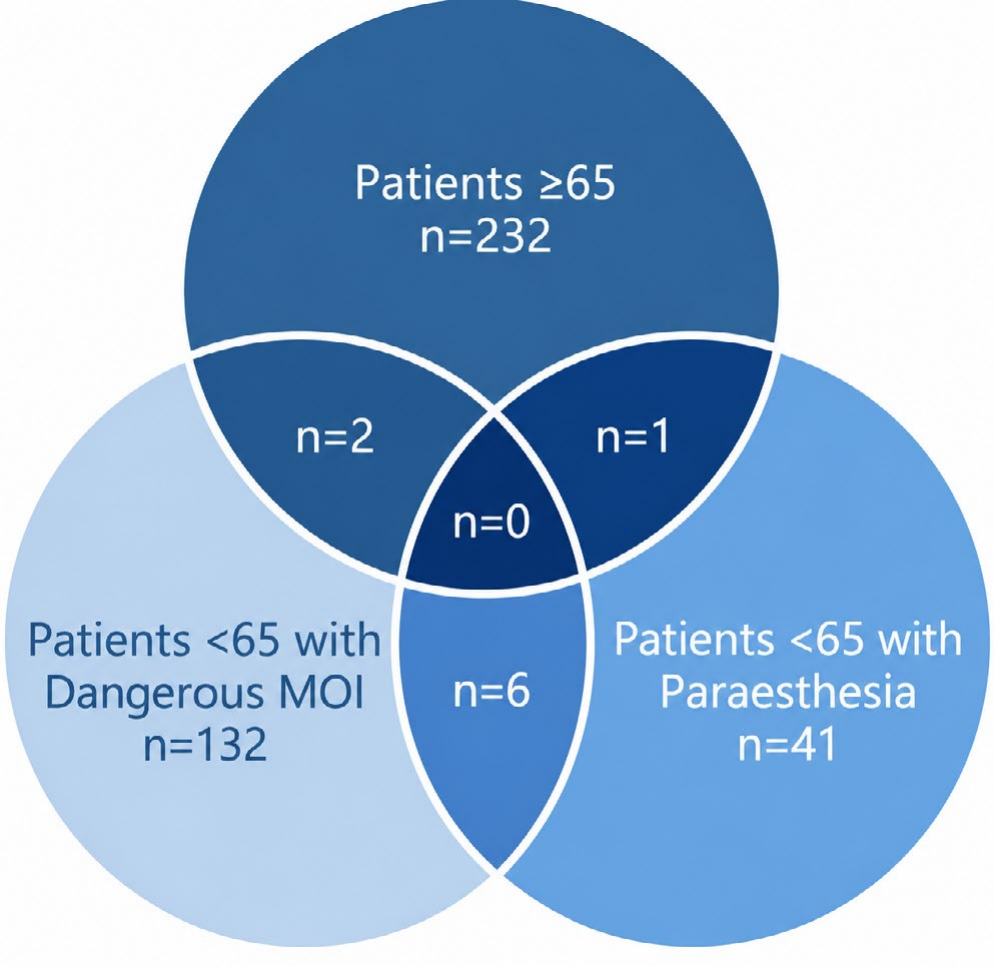
Venn diagram illustrating the reason for categorisation as high‐risk according to NG41 [10].

Overall, 65.5% (*n* = 395/603) of patients were correctly managed and had CT as the initial imaging, including 75.1% (*n* = 311/414) of those categorised as high‐risk based on age, mechanism and/or paraesthesia (Figure 4). Of the remaining patients, the majority (*n* = 116/189; 61.4%) were considered to be low‐risk, but had either a head injury (*n* = 86) or inability to rotate the neck to 45 degrees (*n* = 30). Of the 30 patients who reportedly were unable to actively rotate their neck, 12 (40.0%) had CT initially (one was abnormal), with five having CT following initial X‐rays. Of the low‐risk patients who also presented with a head injury (*n* = 72/86; 83.7%), had CT as the initial imaging; the remaining 14 had X‐ray with one undergoing a subsequent CT due to inadequate X‐rays. The remaining patients (*n* = 73/189; 12.1%) had no risk factors documented in the ED notes or on the imaging referral; the average age of these patients was 37.4 years, most commonly ≤ 40 years of age (*n* = 43/73; 58.9%).

On review of all entries made in the ED and RIS electronic records by the clinician or radiographer involved in the patient's care, all those detailing entries regarding the imaging referral or justification are included in Table 1. This highlighted inconsistencies in ED decision‐making and advice regarding the referral by CT radiographers.

**TABLE 1:** Modality choice influences identified from ED and Radiology records and analysis.

Patient details—mechanism	Verbatim extract	Analysis
ED comments		
23‐year‐old male—high‐risk; MVA	‘*Plan: as advised by ED Consultant for immobilisation and CT whole spine. CT Radiographers called back and advised x‐ray. If concerns then for CT*’	CT radiographer advised inappropriate imaging plan (Patient had X‐rays and subsequent CT scan)
90‐year‐old male—high‐risk; fall	‘*Plan: CT spine*’	Referrer requested X‐ray despite plan being for CT following the primary survey. Patient had CT following X‐ray
17‐year‐old male—high‐risk; MVA	‘*Discussed with ED Consultant—No altered neurology, delayed onset neck pain, self‐extricated, young patient, considering ALARP (as low as reasonably practicable) principle. XR and review*’	Different ED Consultant then advised the referring ANP (advanced nurse practitioner) that the patient required CT ‘*Pt reviewed—advised CT head, C‐spine thorax, abdo, pelvis*’. Patient had a subsequent CT
49‐year‐old male—high‐risk; MVA (Car rollover)	‘*As per ED Consultant X‐ray C‐spine*’	Patient had a subsequent CT
22‐year‐old female—high‐risk; MVA	‘*D/W ED SpR, advised CT C spine due to significant mechanism of Injury. CT called to inform that patient would need a high dose of radiation for the procedure due to her young age and advised X‐ray*’	CT radiographer advised inappropriate imaging plan (Patient had X‐rays but no CT)
32‐year‐old female—high‐risk; fall	‘*Initially planned for cervical CT but CT radiographer advised to do X ray first*’	CT radiographer guided inappropriate imaging plan (X‐ray then a subsequent CT)
61‐year‐old female—low‐risk; MVA	‘*According to Canadian c spine rule: no need for radiography discussed with ED Cons: advised for c spine x‐ray, if NAD (no abnormality detected) discharge*’	Unjustified request but performed
24‐year‐old female—high risk; fall > 2 m	‘*Escalated to CT Radiographer that request made, and discussed 10 min after. The CT night team said the day team will scan the patient. Spoke with the day team who have booked patient in for 09:30, I explained this was inappropriate because the patient will be laying supine for 2 h to which the radiographer questioned why patient could not have an X‐RAY. I explained the Dr has made her clinical judgement using the national guidelines and if they would like to contact her for the discussion she would be happy to speak to them*’	Delay in imaging. CT requested at 07:26, enquiry made at 07:36. Shift handover 08:00–08:30. Patient scanned at 09:52
Radiographer comments		
49‐year‐old male—high‐risk; fall > 2 m	‘*Trauma patient—needs to go to CT. ED nurse informed and porters taken patient back for Doctor to make a new request for CT*’	Radiographer identified X‐ray as unjustified, a further referral for X‐ray was made and performed. The patient had a CT Head examination prior to the X‐ray however no subsequent CT C‐spine
44‐year‐old female—low‐risk MVA (minor rear‐end)	‘*CT Scanner Out of Action*’	ED notes state, ‘*no CT available therefore for c spine x‐ray*’ Unfortunately, the X‐rays were inadequate. No subsequent CT performed even though CT was available at another site within the Trust

**FIGURE 5:**
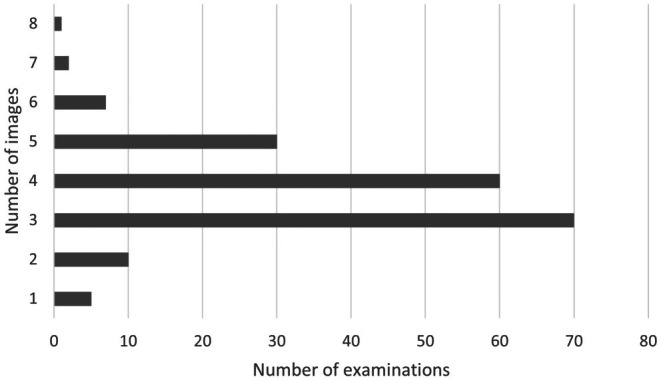
The number of images per X‐ray examination.

The radiation dose for the C‐spine X‐rays was available for half of the patients (*n* = 93/185; 50.3%), the remainder had a cumulative dose for multiple examinations (*n* = 88) or none recorded (*n* = 4). The mean DAP for the examination, inclusive of rejected images, was 52.59cGycm^2^ (range 4.28 cGycm^2^ to 375.00 cGycm^2^), the lowest dose an incomplete examination with only one image. The total number of images released to the PACS varied (Figure 5) with six patients having supplementary projections, including obliques (*n* = 5) and swimmers (*n* = 1). For the examinations with a complete series of projections, including repeats (*n* = 82/185), the average DAP was 54.73 cGycm^2^.

The CT dose was not available for three patients. For patients with CT as the initial imaging investigation (*n* = 415) the mean DLP was 436.8 mGym. Review of those who had both X‐rays and CT scan (*n* = 39) found missing dose data for the CT (*n* = 1) or X‐ray (*n* = 1) and a number of the X‐ray referrals had a cumulative DAP recorded. Therefore, data were only available for 15 patients who had both modalities (Table 2).

**TABLE 2:** Dose and cumulative effective dose for the patients with individual doses recorded (*n* = 15).

Patient	X‐ray DAP (cGycm^2^)	Est. X‐ray effective dose (mSv)	CT DLP (mGycm)	CT effective dose (mSv)	Cumulative effective dose (mSv)
1	22.46	0.035	392	2.313	2.348
2	24.52	0.038	437	2.578	2.616
3	26.97	0.042	388	2.289	2.331
4	28.4	0.044	442	2.608	2.652
5	30.54	0.047	322	1.900	1.947
6	32.65	0.051	236	1.392	1.443
7	39.45	0.061	566	3.339	3.4
8	45.1	0.070	509	3.003	3.073
9	46.2	0.071	539	3.180	3.251
10	48.24	0.075	535	3.157	3.232
11	49	0.076	316	1.864	1.94
12	50.2	0.078	410	2.419	2.497
13	70.12	0.109	410	2.419	2.528
14	117.26	0.182	403	2.378	2.56
15	375	0.581	503	2.968	3.549

Abbreviations: DAP, dose area product, transcribed verbatim from the RIS record and therefore decimal places vary; DLP, dose length product.

Regarding image quality, only 39.5% of X‐rays (*n* = 73/185) were deemed adequate. Of those considered inadequate this was predominantly due to non‐visualisation of the cervical‐thoracic junction (*n* = 75/112; 67.0%). There were also missing projections (*n* = 22/112; 19.6%), suboptimal peg projection (*n* = 9/112; 8.0%), artefacts (*n* = 5/112; 4.5%) and a single examination (0.9%) excluded the upper C‐spine. Comparison of age and image quality found that inadequate examinations represented an older age profile (adequate average 36 years vs. inadequate average 48 years; *z* = −3.630; *p* < 0.05). No significant link was found between gender and cervico‐thoracic junction visualisation (*χ* ^2^ = 0.200, *p* = 0.655), whereas the anatomy was more likely to be seen when patients were not immobilised (*χ* ^2^ = 20.05; *p* < 0.05).

According to the referral information, 53.8% of those who were considered to have inadequate X‐rays went on to have CT (*n* = 21/39). Yet, on independent review 74.4% of the X‐rays (*n* = 29/39) were identified as inadequate. Of the 10 adequate examinations with subsequent CT imaging, nine were categorised as high‐risk. The other had CT despite X‐rays being adequate and low‐risk of a SCI.

The mean reporting turnaround time (TAT) for the X‐ray examinations was 35 h 25 min (ranging from 2 min to 12 days, 14 h and 57 min), whereas for CT it was 1 h 9 min (ranging from 4 min to 25 h and 5 min). Two‐thirds of the X‐ray examinations were normal (*n* = 125/185; 67.6%), the rest demonstrated a trauma‐related abnormality (*n* = 3/185; 1.6%), were inconclusive (*n* = 53/185; 28.6%) or did not have an interpretation documented (*n* = 4/185; 2.2%). The latter were all reported as, for example, ‘Please see the subsequent CT scan report’. Inconclusive reports included those with suboptimal visualisation of anatomy, missing projections and multiple artefacts. Only 16 of the 53 inconclusive X‐ray reports went on to have CT, with one being abnormal: a 73‐year‐old female (high‐risk; fall) who attended on a trolley without immobilisation and had eight X‐ray images taken (AP, lateral, peg and trauma obliques). The X‐rays were deemed inadequate by both the reporter and independent review. Three hours after the X‐ray examination the patient returned fully immobilised for a CT scan which demonstrated ‘Minimally displaced fractures of C1 and C2 vertebrae’. Overall, 4.6% (*n* = 28/603) of the cohort had abnormal imaging findings. A chi‐squared test of independence showed there was no significant relation between the patient gender and the diagnosis (*χ* ^2^ = 0.005, *p* = 0.946). However, abnormal findings were more prevalent in those patients ≥ 61 years (*χ* ^2^ = 9.860 *p* < 0.00169).

## 4 Discussion

This study has reviewed C‐spine imaging performed following trauma in a single organisation, with the inclusion of 1 year of referrals minimising any temporal bias. Further, patient demographics align with previous research [19, 20], thus increasing the reliability of the findings. Although other studies report a younger average age [21, 22, 23], all have notably smaller sample sizes, with two including paediatrics [21, 24] and another excluding CT [24]. Giannakopoulos et al. [25] also reported a younger average age, but they included polytrauma patients. Finally, the Indian study [21] compares to our greater number of females and their longer life expectancy in the UK [25].

Overall, 65.5% of patients were referred straight to CT, with the remainder having either incorrect or unjustified imaging using NG41 [13]. It was speculated, and confirmed, that older patients would be referred for CT with younger patients directed to X‐ray. This is a novel finding and is perhaps only due to the inclusion of both imaging modalities, but does align with the perspective of Benchetrit et al. [18] Importantly, patient age was considered as the sole justification for imaging in 37.9%, aligning with previous research [20]. The injury profile corresponds with published literature, with over 90% of the elderly having fallen [26, 27, 28]. In the younger patients, more men were in the assault, hanging, and sports injury categories [21, 22]. Statistically, more men play sport in all age groups under 65 years [29] and are more likely to commit suicide, with around six out of ten suicides by hanging, strangulation, or suffocation [30]. Additionally, men account for 68.2% of violence‐related ED attendances, with most being younger [31].

The percentage of patients categorised as high‐risk by the CCR is similar to Ngatchou et al. [23]. According to NG41 [13] all patients at high‐risk of C‐spine injury or low‐risk and unable to rotate the neck 45 degrees or with a head injury, require immobilisation. The data demonstrated a statistically significant relationship between patient immobilisation and the imaging modality. National guidelines [9, 13, 32] consider immobilisation to be essential, so the decision not to apply it in 42.7% of X‐ray referrals is controversial, possibly influenced in CT by the need for transfer to the scanner. In our study, immobilisation negatively influenced X‐ray image quality with the main contributors being artefacts and poor visualisation of the lower C‐spine. Interestingly, a recent systematic review concluded that there is a lack of demonstrable benefit to immobilisation [33]. Definitive research is underway [34], recognising the patient safety risks from prolonged immobilisation, with pressure ulcers, raised intracranial pressure and increased optical sheath diameter reported in the literature [7, 35].

In this study the role of the referrer did not influence the choice of modality, although locum doctors and ED consultants were more likely to choose X‐ray over CT. There was also evidence of consultants providing contradictory advice, influencing the inappropriate actions of others. Additionally, a small number of CT referrals were re‐directed to X‐ray by radiographers, resulting in delays and additional radiation dose. NG41 [13] recommendation 1.5.1 states ‘Imaging for spinal injury should be performed urgently’ but in at least one case staff delayed imaging, with shift handover influencing the decision despite ED attempting to expedite the referral.

The number of images varied greatly with over half of X‐ray examinations having repeat or supplementary projections. This is lower than other research [19], perhaps explained by almost 40% of the X‐ray examinations being inadequate, influenced by patient age and immobilisation, similar to Ngatchou et al. [23], with others advocating CT following suboptimal X‐rays [20, 24]. This strengthens the argument against the X‐ray use in C‐spine trauma. Interestingly, Munjal et al. [17] found the adequacy of C‐spine X‐rays prior to, and after, training did not improve the quality. In the 1980s and 90s oblique projections were introduced into the C‐spine series, particularly where the swimmers projection failed to visualise the cervico–thoracic junction [36, 37]. However, the challenges in interpretation [37] and adoption of CT as a diagnostic tool meant they fell out of favour [38]. Undisputedly, the additional X‐rays equated to higher radiation doses, although these did not always result in improved diagnostic value, as 29% of those with repeated projections had inconclusive findings. Importantly, the average DAP for examinations with three images was just below the national dose reference level [39] at 39.82 cGycm2, whereas this was far exceeded by those with four or more images. X‐ray examinations are influenced by a variety of factors, including artefacts, patient position and immobilisation, which all have the potential to increase the DAP. Twenty‐one percent of the patients who initially had X‐rays underwent a subsequent CT, with an average effective dose, where available, of 0.105 mSv. Although this may be considered a relatively low dose, and it is acknowledged there is no safe dose, particularly when imaging is unnecessary or inappropriate [40]. The over‐triage phenomenon whereby resources are used unnecessarily (e.g., the use of X‐rays or CT when not required) [23, 24] has both resource (radiographer and technology) and patient safety implications. Applying the CCR to the ED records, a number of patients were imaged despite no‐risk of C‐spine injury. Others have attempted to address this by implementing a checklist with improved efficacy [35, 41], although this needs to be supported by robust imaging justification processes.

The imaging was normal in 88.7% of patients, slightly less than the 96.4% of Ngatchou et al. [23] but likely influenced by their younger cohort. Patients aged ≥ 61 years were more likely to have an abnormal finding, similar to previous research [1]. Of the 28 patients with abnormal findings, all but one of these patients had CT, with 85.7% as their initial imaging modality; however, four had X‐ray, despite their high‐risk criteria for C‐spine injury. Due to the poor sensitivity and inadequate quality of the X‐ray examinations, each of these patients was perhaps predestined to have CT, and the one who did not had it recommended in the report. According to other NICE guidelines (NG38) [42] ‘A radiologist, radiographer or other trained reporter should deliver the definitive written report of emergency department X‐rays of suspected fractures before the patient is discharged from the emergency department’. In addition, NG41 [13] states ‘…the images should be interpreted immediately by a healthcare professional with training and skills in this area’. With regards to CT, almost all scans were reported within 4 h; however, the X‐ray report TAT fell well short of the expectation that the report should be available prior to the patient being discharged [42].

We conclude that referrers are not consistently employing the CCR when assessing patients, with 12.1% of patients having no risk of C‐spine injury and yet having imaging. Others remark about fear of missing C‐spine fractures and the excessive use of X‐rays [23, 24], and the CCR can reduce unnecessary examinations, but only if actioned appropriately. Ngatchou et al. [23] also allude to patient pressure as a reason for non‐compliance with clinical decision rules; however, we cannot confirm whether this was applicable in our study.

We acknowledge several study limitations. Only a single mechanism of injury was categorised. No attempt was made to corroborate the retrospective data with the individual perspectives of staff regarding appropriate imaging referral and the cause of any delay in imaging or reporting could not be ascertained. The dataset was anonymised and therefore it is unclear whether radiographer experience had any bearing on decision making or radiographic projections performed. The data collection process was time‐consuming, and it is acknowledged that data collection and image analysis was by a single reviewer. Also, the symptoms for those patients having CT as the initial investigation were omitted. Patients who attended with C‐spine trauma and were discharged without imaging were not considered. Furthermore, rejected images were not available; however, the dose recorded was inclusive and the imaging reports were taken as read with no corroborating review. Unfortunately, as this was a retrospective study no body habitus data were available. In addition, many of the DAPs recorded were cumulative, limiting the dose calculations, and it is acknowledged that there is a requirement for dose reporting information systems and people are not infallible. Also, inferences had to be made from the ED notes which were not always detailed, particularly regarding the presenting symptoms. One of the main disadvantages of the retrospective approach is that it cannot conclusively determine why patients had imaging despite it not being indicated by the CCR; it is also recognised that there is no legal requirement for adherence to NICE best practice guidance. Importantly, the data presented is from a single NHS Trust and therefore although the results cannot be generalised, they should provide a baseline and a prompt for others to review their practice.

## 5 Conclusions

In‐depth analysis of a cohort of 603 patients confirmed that in this organisation the majority of patients were having justified and appropriate imaging. However, there was evidence of non‐compliance with national guidance both in terms of risk assessment and utilisation of X‐ray imaging as a primary imaging modality. The results have radiation dose implications related to the inappropriate imaging, along with excessive radiation doses exacerbated by poor quality X‐rays resulting in inconclusive outcomes. The findings also demonstrate varying application of immobilisation, although when CT scans are required a more consistent approach is noted but imaging related delays were observed, resulting in prolonged immobilisation.

This study has confirmed the need for a consistent, evidence‐based approach to the management of patients following C‐spine trauma and a collaborative strategy for assessment and imaging involving all relevant professional groups. It should promote review of clinical practice across specialities and organisations to ensure patient safety and the judicious use of radiation and resources. The findings should also act as a reminder to radiographers of their role in the justification of imaging examinations and the limitations of X‐rays in the investigation of these patients.

## Funding

The authors have nothing to report.

## Ethics Statement

Ethical approval was given by University of Bradford (ref 23039456) and the NHS Trust (ref 0016/2024/RAD/MI/CB).

## Conflicts of Interest

The authors declare no conflicts of interest.

## Data Availability

The data that support the findings of this study are available from the corresponding author upon reasonable request.
